# Seeking quantitative morphological characters for species identification in soldiers of Puerto Rican *Heterotermes* (Dictyoptera, Blattaria, Termitoidae, Rhinotermitidae)

**DOI:** 10.3897/zookeys.725.20010

**Published:** 2017-12-29

**Authors:** Zachary H. Griebenow, Susan C. Jones, Tyler D. Eaton

**Affiliations:** 1 Department of Entomology, The Ohio State University, 2501 Carmack Rd., Columbus, OH, 43210-1065, USA; 2 Department of Entomology & Nematology, University of California-Davis, 381A Briggs Hall, One Shields Ave., Davis, CA, 95616-5270, USA

**Keywords:** Caribbean, *Heterotermes cardini*, *Heterotermes convexinotatus*, *Heterotermes tenuis*, Morphometrics, taxonomy

## Abstract

Subterranean termites in the genus *Heterotermes* Froggatt (Rhinotermitidae: Heterotermitinae) are pantropical wood feeders capable of causing significant structural damage. The aim of this study was to investigate soldier morphological attributes in three Puerto Rican species of *Heterotermes* previously identified by sequencing of two mitochondrial genes and attributed to *Heterotermes tenuis* (Hagen), *H. convexinotatus* (Snyder) and *H. cardini* (Snyder). Soldiers (n = 156) were imaged and measured using the Auto-Montage image-stacking program. We demonstrated that Puerto Rican *Heterotermes* soldiers could not be identified to species level based upon seven morphometric indices or any combination thereof. Nor could differences in soldier head pilosity be used to discriminate species, in contrast to previous findings. However, previously described characters of the soldier tergal setae were reported to be useful in discriminating *H. tenuis* from both of its Puerto Rican congeners.

## Introduction

*Heterotermes* Froggatt, 1897 (Rhinotermitidae: Heterotermitinae) is a pantropical genus of subterranean wood feeding termites (Constantino 2000). Seventeen species have been reported as pests that damage human structures (Scheffrahn and Su 2000). The *Heterotermes* fauna in the Caribbean Region (the Bahamas, Greater Antilles, and Lesser Antilles) is thought to consist exclusively of pest species that have been introduced from the South American mainland (Constantino 1998, Evans et al. 2013). Caribbean *Heterotermes* are consequently of interest in that they are both invasive and economically significant. In the Caribbean Region, the Puerto Rican archipelago is situated at the eastern end of the Greater Antilles and is adjacent to the younger, actively volcanic Lesser Antilles, hence providing a biotic link to the South American mainland.

Our understanding of *Heterotermes* species’ identity and distribution in the Caribbean Region has fluctuated over time, with the species composition of the *Heterotermes* fauna in Puerto Rico and its associated islands being quite ambiguous. Snyder (1956) reported *Heterotermes tenuis* (Hagen, 1858) and *Heterotermes convexinotatus* (Snyder, 1924) from the archipelago, while Scheffrahn et al. (2003) left Puerto Rican *Heterotermes* specimens unidentified to species level due to taxonomic uncertainty. Using a phylogenomic approach conjunct with some morphological and biogeographical data, Szalanski et al. (2004) concluded that *Heterotermes cardini* (Snyder, 1924) was present in the Caribbean Region in addition to *H. tenuis* and *H. convexinotatus*, along with one or more undescribed species. Three samples from Puerto Rico were included in their study, all identified as *H. convexinotatus*. In contrast, Eaton et al. (2016) reported *H. tenuis*, *H. convexinotatus*, and *H. cardini* in Puerto Rico based on molecular phylogenies of two mitochondrial loci from 76 Puerto Rican samples, with morphological confirmation of species identification. Furthermore, their phylogenomic data provided strong evidence that the proposed undescribed *Heterotermes* (Szalanski et al. 2004) were consistent with *H. cardini*.

*Heterotermes* soldiers from the Caribbean remain difficult to reliably distinguish due to non-robust diagnostic morphological characters: Snyder (1924) asserted that soldiers of the three species differed in cephalic and pronotal pilosity, body coloration, and relative size. However, due to morphological ambiguity, Snyder (1924) suggested in his original descriptions of *H. convexinotatus* and *H. cardini* (the latter described from the Bahamas) that they might be synonymous with *H. tenuis*. Consequently, alates (winged reproductives) are essential for reliable species identification of those *Heterotermes* spp. putatively present in Puerto Rico (Snyder 1924; Szalanski et al. 2004; Eaton et al. 2016). Since alates are produced only seasonally, they are difficult to obtain and are seldom properly associated with their parent colonies. Therefore, robust soldier-based identification of Puerto Rican *Heterotermes* is of practical taxonomic interest.

The purpose of our study was to measure morphometric parameters in the soldier caste from a comprehensive sample of the Puerto Rican *Heterotermes* fauna, and to determine what parameters, if any, were most useful in identifying *Heterotermes* to species level. Additionally, pilosity of the head capsule (Snyder 1924) and abdominal tergites of *Heterotermes* soldiers (Constantino 2000) were examined as diagnostic characters. The possibility of an additional, undescribed species of *Heterotermes* in the Caribbean Region (as per Szalanski et al. 2004) was also herein analyzed.

## Materials and methods

Our study is supplementary to that of Eaton et al. (2016) in that it largely uses a subset of the same Puerto Rican *Heterotermes* samples (n = 60 of 76 samples). These were assigned to *H. tenuis*, *H. convexinotatus*, or *H. cardini* on the basis of their 16S rRNA and cytochrome oxidase subunit II (COII) phylogeny. Samples were collected by SCJ from different locales on the main island of Puerto Rico and adjacent Culebra Island in 2002, 2004, 2006, or 2010. Each sample consisted of termites collected from a single access point in a given colony and placed in individual vials filled with absolute alcohol.

**Figure 1.**
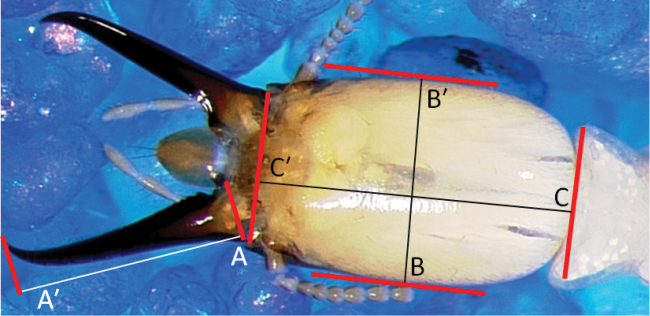
Cephalic metrics: AA' = maximum length of mandible; BB' = maximum width of head; CC' = length of head to lateral base of mandibles. Parallels used to delineate metrics (see Suppl. material 1) are marked in red.

**Figure 2.**
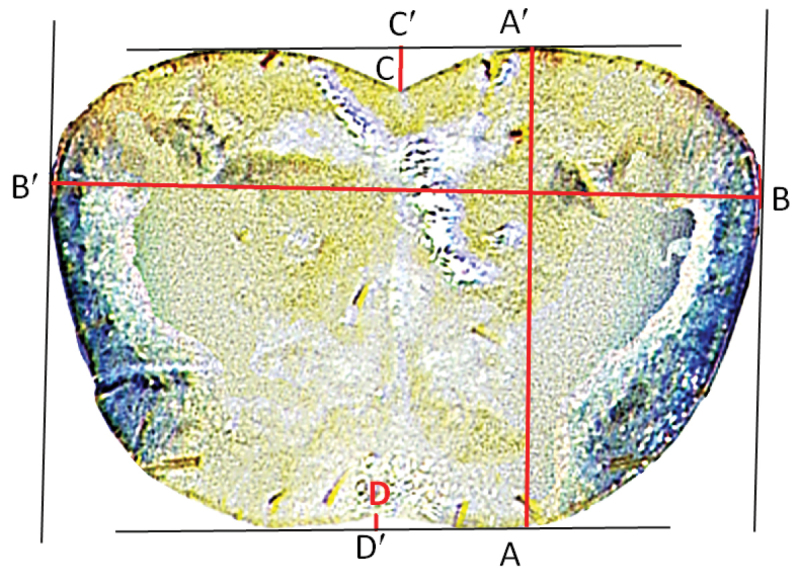
Pronotal metrics: AA'= maximum length of pronotum; BB' = maximum width of pronotum; CC' = depth of anterior pronotal notch; DD' = depth of posterior pronotal notch. Parallels are marked in black.

In total, 156 individual soldiers were examined morphometrically (see Suppl. material 1), with 3 being selected, when available, from each sample, providing 40 specimens of *H. tenuis*, 55 *H. convexinotatus*, and 61 *H. cardini*. We investigated mandible length in combination with the same axis of the head capsule, along with head width (Figure 1), in order to provide additional commonality with the data presented in Constantino (2000) and Szalanski et al. (2004). In addition, multiple pronotal metrics (Figure 2) were examined, two of them novel to this study (see Suppl. material 1). A total of three morphometric indices recommended by Roonwal (1969) for use in termite taxonomy were derived from a subset of these metrics (see Suppl. material 1). Cephalic setae were surveyed in 79 of the 156 soldiers, plus an additional 7 *H. convexinotatus* soldiers examined without morphometric analysis. Also, setae within a 300-μm radius of the soldier fontanelle were censused in 66 of the 156 soldiers. A subset of 45 of the 156 soldiers was used for characterization of tergal setae.

A Z16 AP0 stereomicroscope (Leica Microsystems, Buffalo Grove, USA) with a KY-570B camera (JVC, Wayne, USA) was used to image specimens for morphometric analysis and examination of tergal setae, with images being stacked into montage microphotographs using Auto-Montage Pro software (ver. 5.01.0005, Synoptics Ltd., Cambridge, UK).

We performed analyses of variance (ANOVA), discriminant analyses, and *k*-means cluster analyses on morphometric data using SPSS Statistics 24 (2016, International Business Machines, Armonk, USA). The level of significance for all analyses was set at α=0.05. Based on the results of ANOVA, a discriminant analysis and three *k*-means cluster analyses were performed using all statistically significant parameters. Discriminant analyses determine the efficacy of any array of variables in assigning group membership (Green et al. 2008).

*K*-means cluster analyses do not assume *a priori* assignments of specimens to groups, instead attempting to iteratively delineate groups *de novo* from the data given according to the number of groups provided by an *a priori* hypothesis. Our *k*-means cluster analyses specified 3, 4, and 2 morphometric-delineated groups within the sampled *Heterotermes* fauna. The first analysis was premised according to the conclusion of Eaton et al. (2016) that there are 3 valid *Heterotermes* spp. in the Puerto Rican archipelago. The second was premised according to the conclusion of Szalanski et al. (2004) that 4 *Heterotermes* spp. occur in the Caribbean Region. The third was premised according to the phylogenetic hypothesis that *H. tenuis* is sister to a clade including *H. convexinotatus* and *H. cardini* (Szalanski et al. 2004; Eaton et al. 2016): if this were the case, one would expect specimens to group into two clusters. It was not possible to delineate one or more metrics on a total of 40 specimens (5 *H. tenuis*, 8 *H. convexinotatus*, and 27 *H. cardini*), so these were excluded from *k*-means cluster analysis.

## Results and discussion

ANOVA concerning all parameters demonstrated that all but one of the metrics surveyed – the depth of the posterior pronotal notch, which was novel to this study – displayed statistically significant variation across all three species (Table 1). Only one of the three morphometric indices we derived – the head-mandible index (Roonwal 1969) – exhibited statistically significant variation across all three species (Table 1).

**Table 1.** Results of an ANOVA assessing variation of select morphometric parameters. Parameters exhibited statistically significant differentiation if *P*<0.05.

Parameter	df	*F* value	*P* value
Head capsule length	127	36.089**	0.000
Mandible length	124	36.089**	0.000
Head width	126	50.033**	0.000
Pronotum width	150	46.723**	0.000
Pronotum length	150	15.759**	0.042
Depth of anterior pronotal notch	146	3.242**	0.000
Depth of posterior pronotal notch	122	42.885	0.826
Pronotal index	150	0.575	0.564
Head-mandible index	124	6.732**	0.002
Head index	127	1.434	0.242

Discriminant and *k*-means cluster analyses used only statistically significant parameters. Of all 116 specimens with sufficient morphometric data for their inclusion in discriminant analysis, our discriminant analyses correctly classified 87.1% to species. Thus, while the majority of Puerto Rican *Heterotermes* with sufficient morphometric data could be accurately identified to species level using statistically significant morphometric parameters, these data were not invariably reliable for that purpose; nor was this mode of identification concise given that a total of seven parameters was necessary.

The results of morphometric 3- and 4-cluster analyses did not consistently conform to the phylogenetic hypotheses of Eaton et al. (2016) and Szalanski et al. (2004) for Caribbean *Heterotermes*, respectively, nor did any cluster analysis support placement of *H. tenuis* as a sister-group to the remaining two putative species of *Heterotermes*. Furthermore, no morphometric consensus, novel or otherwise, could be made across all three *k*-means cluster analyses (Table 2). These results were likely influenced by the fact that many specimens (n = 40) provided insufficient data to be included in cluster analyses.

**Table 2.** Cluster membership under respective phylogenetic hypotheses. Insufficient morphometric data precluded analysis of 40 specimens. Cluster numeration was arbitrary. Distance was measured relative to computed cluster center.

Species	2-Cluster Hypothesis		3-Cluster Hypothesis		4-Cluster Hypothesis	
	Cluster	Distance	Cluster	Distance	Cluster	Distance
*H. tenuis*	2	253.54143	3	32.20008	3	46.50913
*H. tenuis*	2	326.77099	3	74.86261	3	53.2888
*H. tenuis*	2	354.21671	3	117.6952	3	93.6614
*H. tenuis*	2	85.55526	2	84.16529	2	81.85684
*H. tenuis*	2	312.57804	3	87.58546	3	83.58083
*H. tenuis*	2	309.6662	3	84.9422	3	85.65045
*H. tenuis*	2	256.37002	3	39.99476	3	57.39513
*H. tenuis*	2	234.62472	3	81.46453	3	100.08422
*H. tenuis*	2	121.91754	2	103.66997	2	106.89786
*H. tenuis*	2	251.06997	3	52.89946	3	68.95685
*H. tenuis*	2	314.68978	3	61.76796	3	41.12887
*H. tenuis*	1	160.0722	1	163.05486	1	163.73504
*H. tenuis*	2	229.03476	3	62.02824	3	85.69223
*H. tenuis*	2	340.18453	3	86.72816	3	58.86668
*H. tenuis*	2	347.37142	3	86.32921	3	62.79373
*H. tenuis*	2	165.30565	2	233.59069	2	169.00256
*H. tenuis*	2	113.2921	2	132.59409	2	102.7966
*H. tenuis*	2	314.97147	3	66.84973	3	43.15213
*H. tenuis*	2	108.07079	2	186.68133	2	125.18157
*H. tenuis*	2	208.05716	3	109.93034	3	128.41514
*H. tenuis*	2	310.24474	3	73.71241	3	55.89708
*H. tenuis*	2	124.9129	3	142.88123	2	146.78559
*H. tenuis*	2	380.73388	3	124.49842	3	99.2555
*H. tenuis*	2	166.58084	3	234.09751	2	177.17161
*H. tenuis*	2	326.48907	3	68.58936	3	46.22216
*H. tenuis*	2	151.22401	2	67.91019	4	91.83434
*H. tenuis*	2	193.01248	2	85.87651	4	43.17484
*H. tenuis*	1	282.70386	2	194.37339	4	102.57221
*H. tenuis*	2	264.30373	2	154.32751	4	69.74912
*H. tenuis*	2	230.22493	2	126.27088	4	65.41226
*H. tenuis*	1	301.11995	2	209.09383	4	124.68136
*H. tenuis*	2	267.73784	2	157.77828	4	82.99303
*H. tenuis*	1	301.10911	2	206.37768	4	120.70212
*H. tenuis*	2	280.63396	2	180.37562	4	113.16429
*H. tenuis*	2	57.2989	2	166.99186	2	76.70921
*H. convexinotatus*	2	66.64623	2	162.67921	2	76.51109
*H. convexinotatus*	2	100.58655	2	74.61512	2	87.54042
*H. convexinotatus*	2	45.53643	2	73.81509	2	30.55171
*H. convexinotatus*	1	92.8697	1	106.97731	1	109.89883
*H. convexinotatus*	1	42.58346	1	61.12458	1	66.23971
*H. convexinotatus*	1	48.37568	1	68.29212	1	72.47772
*H. convexinotatus*	1	170.71652	1	197.58664	1	201.95907
*H. convexinotatus*	1	100.92468	1	129.95624	1	134.78135
*H. convexinotatus*	1	195.08743	1	171.49698	1	168.40471
*H. convexinotatus*	1	143.58385	1	159.21383	1	161.85759
*H. convexinotatus*	1	39.48472	1	61.0434	1	65.59095
*H. convexinotatus*	2	190.79254	2	87.65711	4	74.07688
*H. convexinotatus*	1	131.65924	1	160.45317	1	165.14755
*H. convexinotatus*	1	147.95901	1	173.5811	1	177.67905
*H. convexinotatus*	2	100.109	2	88.36581	2	85.5312
*H. convexinotatus*	2	256.96346	2	159.35016	4	111.01812
*H. convexinotatus*	1	63.17991	1	65.26824	1	67.17037
*H. convexinotatus*	1	172.68076	1	197.98883	1	201.96107
*H. convexinotatus*	1	89.89609	1	79.38557	1	79.56232
*H. convexinotatus*	2	219.01414	2	121.76263	4	78.87236
*H. convexinotatus*	1	115.93995	1	116.1732	1	117.93494
*H. convexinotatus*	1	61.6565	1	39.65379	1	39.16278
*H. convexinotatus*	2	161.9857	2	71.84719	4	93.15414
*H. convexinotatus*	2	168.94894	2	67.60631	4	72.92106
*H. convexinotatus*	2	72.73062	2	96.75358	2	55.58775
*H. convexinotatus*	2	98.61692	2	51.1218	2	75.0215
*H. convexinotatus*	2	76.54337	2	152.03536	2	76.50752
*H. convexinotatus*	2	109.20261	2	40.35267	2	86.98483
*H. convexinotatus*	2	73.69146	2	144.78302	2	80.01889
*H. convexinotatus*	2	230.57425	2	133.95943	4	100.72459
*H. convexinotatus*	2	107.73092	2	119.16952	2	90.72008
*H. convexinotatus*	2	146.14797	3	189.50569	2	156.36624
*H. convexinotatus*	2	130.50162	2	169.64244	2	130.77036
*H. convexinotatus*	2	239.38575	2	139.56988	4	99.8207
*H. convexinotatus*	2	92.55724	2	120.63307	2	77.22261
*H. convexinotatus*	2	116.67981	2	53.59368	2	94.82665
*H. convexinotatus*	2	143.72191	2	63.84648	4	102.7939
*H. convexinotatus*	2	55.07361	2	164.29003	2	72.06005
*H. convexinotatus*	2	100.55303	2	135.18375	2	88.91133
*H. convexinotatus*	2	116.60445	2	73.3531	2	94.07228
*H. convexinotatus*	2	82.23475	2	103.79059	2	65.28595
*H. convexinotatus*	2	160.4155	2	85.86284	4	111.32346
*H. convexinotatus*	2	89.50309	2	187.87274	2	101.76863
*H. convexinotatus*	2	167.55718	2	109.25237	4	137.91008
*H. convexinotatus*	2	79.39547	2	121.25412	2	71.32581
*H. convexinotatus*	2	124.51904	2	119.70772	2	123.62927
*H. convexinotatus*	2	101.38499	2	44.47996	2	80.3574
*H. cardini*	1	50.62172	1	49.4603	1	52.54455
*H. cardini*	2	156.96056	2	72.06053	4	92.09658
*H. cardini*	2	116.3222	2	68.96384	2	105.05938
*H. cardini*	2	133.27853	2	58.6334	4	107.50376
*H. cardini*	1	69.85903	1	50.30866	1	49.75939
*H. cardini*	1	63.33121	1	60.63669	1	62.57799
*H. cardini*	1	228.32884	2	242.53297	4	151.5971
*H. cardini*	1	233.92987	2	224.04711	4	131.26097
*H. cardini*	1	152.27189	1	122.78914	1	118.84265
*H. cardini*	1	156.08061	1	133.17036	1	130.72768
*H. cardini*	1	127.96476	1	98.93537	1	95.34782
*H. cardini*	1	186.62386	1	223.11212	4	204.59382
*H. cardini*	1	187.26549	1	157.73427	1	153.65728
*H. cardini*	1	183.74985	1	157.39755	1	154.03872
*H. cardini*	1	233.73398	1	198.98527	1	193.37452
*H. cardini*	1	74.31731	1	92.99662	1	96.63796
*H. cardini*	1	103.83294	1	105.39398	1	107.29047
*H. cardini*	1	217.06493	1	192.74338	1	189.6661
*H. cardini*	1	201.84888	1	171.83761	1	167.68972
*H. cardini*	1	238.91474	1	207.60536	1	203.09527
*H. cardini*	1	195.04476	1	163.03509	1	158.21636
*H. cardini*	2	150.4453	2	93.68494	4	126.39925
*H. cardini*	2	135.8617	2	70.67088	4	111.95337
*H. cardini*	2	164.19757	2	112.11675	4	137.5087
*H. cardini*	2	279.99234	2	196.786	4	155.38894
*H. cardini*	1	215.87616	1	214.5414	1	214.2815
*H. cardini*	1	289.80952	1	272.98807	1	269.8637
*H. cardini*	2	141.99891	2	98.41767	2	134.63763
*H. cardini*	2	178.27138	2	79.26567	4	61.65641
*H. cardini*	2	156.7643	2	92.67277	4	116.96033
*H. cardini*	2	106.70269	2	86.74184	2	103.08692
*H. cardini*	2	93.76761	2	142.89509	2	106.73515
*H. cardini*	1	66.89762	1	65.16361	1	66.45667
*H. cardini*	1	178.07806	1	183.42556	1	185.36444

We found short to medium-length (uniformly <100 μm) setae on the head capsules of all *H. convexinotatus* specimens examined with respect to this character, but pilosity of this species and *H. tenuis* was comparable (Table 3). Furthermore, there was considerable overlap in head capsule pilosity among all three species (Table 4). Likewise, the area within a 300-μm radius of the fontanelle of a subset of the 156 specimens displayed considerable overlap in seta quantity and no apparent pattern among all three species (Table 5). We therefore conclude that soldier head pilosity is not a reliable character for discriminating among Puerto Rican *Heterotermes* species, contrary to Snyder (1924).

**Table 3.** Summary statistics for cephalic seta counts.

Species	Number of soldiers	Number of samples	Average	Standard error
*H. tenuis*	13	2	18.6923	2.48427
*H. convexinotatus*	36	15	16.6111	0.9818
*H. cardini*	37	13	14.1081	0.85053

**Table 4.** Cephalic setae counts. Identifiers are as follows: sample #_year of collection_replicate_species.

Identifier	# of cephalic setae
67_10_01_tenuis	27
84_06_01_tenuis	26
84_06_02_tenuis	25
84_06_03_tenuis	25
84_06_04_tenuis	29
84_06_05_tenuis	2
84_06_06_tenuis	10
84_06_07_tenuis	16
84_06_08_tenuis	22
84_06_09_tenuis	19
84_06_10_tenuis	26
84_06_11_tenuis	8
84_06_12_tenuis	8
235_04_01_convexinotatus	19
34_06_01_convexinotatus	13
66_06_01_convexinotatus	15
226_04_01_convexinotatus	20
226_04_02_convexinotatus	13
226_04_03_convexinotatus	21
233_04_01_convexinotatus	27
233_04_02_convexinotatus	17
233_04_03_convexinotatus	26
74_02_01_convexinotatus	18
74_02_02_convexinotatus	17
74_02_03_convexinotatus	21
64_02_01_convexinotatus	12
64_02_02_convexinotatus	19
64_02_03_convexinotatus	15
257_04_01_convexinotatus	10
257_04_02_convexinotatus	23
257_04_03_convexinotatus	16
267_04_01_convexinotatus	16
267_04_02_convexinotatus	25
267_04_03_convexinotatus	25
258_04_01_convexinotatus	17
258_04_02_convexinotatus	24
258_04_03_convexinotatus	17
427_04_01_convexinotatus	11
427_04_02_convexinotatus	14
427_04_03_convexinotatus	20
414_04_02_convexinotatus	2
404_04_01_convexinotatus	14
407_04_01_convexinotatus	7
407_04_02_convexinotatus	14
407_04_03_convexinotatus	5
407_04_04_convexinotatus	8
428_04_01_convexinotatus	18
428_04_02_convexinotatus	16
428_04_03_convexinotatus	23
10_02_01_cardini	18
10_02_02_cardini	13
218_04_01_cardini	9
218_04_02_cardini	11
218_04_03_cardini	6
22_02_01_cardini	15
22_02_02_cardini	19
22_02_03_cardini	19
222_04_01_cardini	15
222_04_02_cardini	14
222_04_03_cardini	15
25_06_01_cardini	13
25_06_02_cardini	13
25_06_03_cardini	8
31_06_01_cardini	4
32_02_01_cardini	16
32_02_02_cardini	20
32_02_03_cardini	18
44_02_01_cardini	6
44_02_02_cardini	14
44_02_03_cardini	5
65_02_01_cardini	10
65_02_02_cardini	20
65_02_03_cardini	20
66_02_01_cardini	14
66_02_02_cardini	8
66_02_03_cardini	11
67_02_01_cardini	16
67_02_02_cardini	21
67_02_03_cardini	14
78_02_01_cardini	17
78_02_02_cardini	18
78_02_03_cardini	9
79_02_01_cardini	9
79_02_02_cardini	20
79_02_03_cardini	26
79_02_04_cardini	18

**Table 5.** Summary statistics for seta counts within a 300-µm radius of the soldier fontanelle.

Species	Number of soldiers	Number of samples	Average	Standard error
*H. tenuis*	13	2	3.184211	0.190053
*H. convexinotatus*	15	7	5.666667	0.214214
*H. cardini*	38	14	4.230769	0.405096

**Figure 3.**
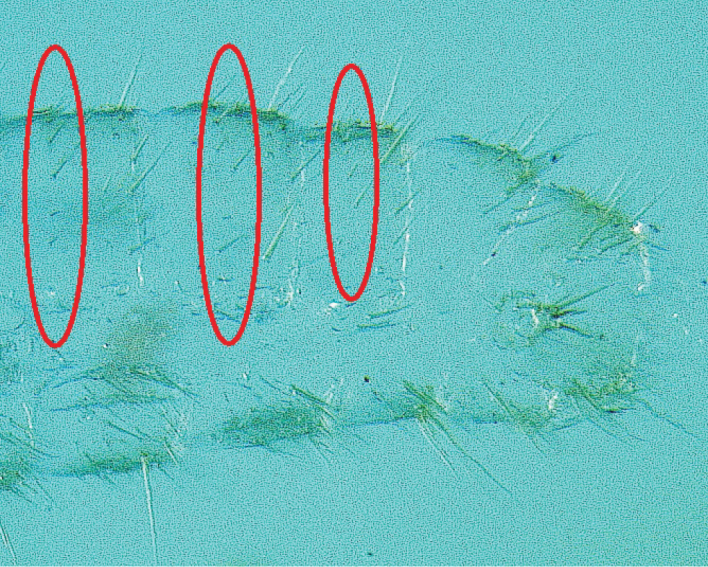
Posterior abdominal tergites of *H. tenuis*. Rows of long setae on central tergal surfaces are circled.

Although soldiers of all three *Heterotermes* species bore a distinct line of bristles (setae noticeably longer than surrounding setae) along the posterior margins of abdominal tergites (excluding the epiproct), only *H. tenuis* soldiers had a distinct row of long hairs on central tergal surfaces (Figure 3). In contrast, short setae (often <10 μm long) not arranged in distinct rows predominated on the central tergal surfaces of *H. convexinotatus* and *H. cardini* soldiers. These correspond to the “numerous microscopic hairs” described by Constantino (2000) on *H. convexinotatus* tergites. Whereas Constantino (2000) did not compare the tergal pilosity of *H. cardini* to *H. tenuis*, we make a novel report that tergal seta distribution also can be used to distinguish *H. tenuis* soldiers from those of *H. cardini*.

## Conclusions

Our findings demonstrated that examined morphometric characters of the soldier pronotum and head could not reliably differentiate *Heterotermes* species in the Puerto Rican archipelago. These data did not provide any well-resolved distinction between the three putative species in question, such as is supported by rigorous phylogenomic investigation (Eaton et al. 2016). We also found that *H. convexinotatus* and *H. cardini* soldiers could not be unequivocally discriminated by cephalic seta counts, contrary to Snyder (1924). However, *H. tenuis* soldiers could be readily identified by means of tergal setal distribution, as reported by Constantino (2000). We furthermore report that soldier tergal seta distribution is useful to distinguish *H. tenuis* from *H. cardini*.

## Supplementary materials

Supplementary material 1 Definitions of soldier morphometric parameters Data type: taxonomic metrics and indices. Explanation note: Definitions of all morphometric parameters utilized in this study, *sensu* Roonwal (1969). This is an open access article distributed under the terms of the Creative Commons Attribution License (CC BY 4.0), which permits unrestricted use, distribution, and reproduction in any medium, provided the original author and source are credited.Zachary H. Griebenow, Susan C. Jones, Tyler D. Eaton
