# Supplementary material for: Seeking quantitative morphological characters for species identification in soldiers of Puerto Rican Heterotermes (Dictyoptera, Blattaria, Termitoidae, Rhinotermitidae)
Source: Zookeys. 2017 Dec 29;(725):17–29. doi: 10.3897/zookeys.725.20010 (PMC5769715; doi:10.3897/zookeys.725.20010)
Supplement: Supplementary material 1 — Definitions of soldier morphometric parameters [file zookeys-725-017-s001.pdf]

Definitions of Soldier Morphometric Parameters

The following metrics were delineated on each soldier image whenever possible:

1) Maximum length of mandible (Fig. 1, line AA')—defined as the distance between two parallels marking the notch at the upper base of the outer mandibular condyle and the distalmost tip of the mandible (i.e., not measured diagonally across the mandible from the condyle to the mandible's inner tip);
2) Maximum width of head (Fig. 1, BB'—distance between two parallels marking the outermost lateral margins of the head capsule;
3) Length of head to lateral base of mandibles (Fig. 1, CC'; effectively synonymous in the case of *Heterotermes* with "head-capsule length" as defined by Roonwal 1969)—defined as the distance between two parallels marking the hindmost margin of the head-capsule and the external articulations of the mandibles;
4) Maximum pronotal length (Fig. 2, AA')—distance between two parallels marking the foremost and hindmost margins of the pronotum at their widest separation;
5) Maximum pronotal width (Fig. 2, BB')—distance between two parallels marking the lateral margins of the pronotum at their widest separation.

In addition, two novel metrics were used:

1) Depth of the anterior pronotal notch (Fig. 2, CC'), defined as the distance between the hindmost extent of the anterior pronotal margin and a parallel tangential to both vertices of the anterior pronotal margin;
2) Depth of the posterior pronotal notch (Fig. 2, DD'), defined as the distance between the foremost extent of the posterior pronotal margin and a parallel tangential to both vertices of the posterior pronotal margin.

From a subset of these metrics, the following indices (as defined by Roonwal 1969) were derived for statistical analysis in addition to the above-iterated metrics:

1) Mandible-head index I—defined as $\frac{Maximum\ length\ of\ mandible}{Length\ of\ head\ to\ lateral\ base\ of\ mandibles}$;

2) Head index I—defined as $\frac{Maximum\ width\ of\ head\ with\ eyes\ (when\ present)}{Length\ of\ head\ to\ lateral\ base\ of\ mandibles}$;

3) Pronotum index I—defined as $\frac{Maximum\ length\ of\ pronotum}{Maximum\ width\ of\ pronotum}$.
